# People, places and the climate emergency – the Scottish Place Standard Tool with a climate lens

**DOI:** 10.1093/eurpub/ckac129.359

**Published:** 2022-10-25

**Authors:** K Hasler, S Whitmore, R Wolstenholme, C Payne, A Hutton, C Fingland, G Tarvit

**Affiliations:** 1 Division of Architecture and Planning, Scottish Government, Glasgow, UK; 2 Public Health Scotland, Glasgow, UK; 3 Sniffer, Edinburgh, UK; 4 Architecture & Design Scotland, Edinburgh, UK; 5 Sustainable Scotland Network, Edinburgh, UK

## Abstract

Since the launch of the Place Standard tool (PST) in Scotland in 2015 awareness has increased of the critical impact of the climate emergency on health and equity. A 2019 review of the PST, informed by emerging evidence and community and stakeholder feedback, confirmed the need to strengthen its contribution towards place-based climate action. This was partly achieved by integrating enhanced prompts within the PST itself, however with the increased focus of policy and action around climate adaptation and mitigation a knowledge and resource gap remained. So in 2020 PST partners* began work with experts from environmental organisations (Sniffer, Sustainable Scotland Network) and other partners to develop a “Place Standard with a climate lens” (PST CL). The PST CL toolkit was created through an iterative process integrating feedback from 10 pilot projects chosen to represent the varied communities, scales, landscapes and placemaking projects being undertaken across Scotland. It provides a suite of materials to use alongside existing PST resources to help placemaking conversations consider how climate change might play out in a local area. This ensures that local responses to climate change are designed holistically, delivered collaboratively, and helps achieve on other local priorities such as health, wellbeing and equity. This presentation will provide a brief overview of the project background, describe the Scottish PST with a climate lens, share case study examples from the piloting phases, and enable exploration of the learning from Scotland around the value of integrating health and climate in place-based approaches. While the Place Standard tool was not originally designed as a climate change tool, it is an effective method to support the design of local responses to the climate emergency. A “climate lens” can help us to plan the future of our places to maximise the health benefits and minimise the negative consequences of a changing climate.

